# Helios characterized circulating follicular helper T cells with enhanced functional phenotypes and was increased in patients with systemic lupus erythematosus

**DOI:** 10.1007/s10238-023-01289-6

**Published:** 2024-01-19

**Authors:** Xingyue Zeng, Xiayidan Alimu, Ayibaota Bahabayi, Zhonghui Zhang, Mohan Zheng, Zihang Yuan, Tianci Liu, Chen Liu

**Affiliations:** 1 Department of Clinical Laboratory, Peking University People’s Hospital, 11# Xizhimen South Street, Beijing, 100044 China; 2 School of Basic Medical Sciences, Peking University Health Science Center, Beijing, China

**Keywords:** IKZF2, Helios, TFH, TFR, SLE

## Abstract

Helios was related to the immunosuppressive capacity and stability of regulatory T cells. However, the significance of Helios in follicular help T (TFH) and follicular regulatory T (TFR) cells is unclear. This research aimed to clarify the significance of Helios (*IKZF2*) in TFH and TFR cells and its clinical value in systemic lupus erythematosus (SLE). *IKZF2* mRNA in different cell subsets was analyzed. Helios+ percentages in TFH and TFR cells were identified in the peripheral blood of 75 SLE patients and 62 HCs (healthy controls). PD-1 and ICOS expression were compared between Helios+ and Helios− cells. The capacity of TFH cells to secrete IL-21 and TFR cells to secrete IL-10 was measured. Correlation analysis and receiver operating characteristic (ROC) curve analysis were conducted to assess the clinical significance of Helios-related TFH and TFR cell subsets in SLE. There was Helios expression in TFH and TFR cells. PD-1 and ICOS were lower in Helios+ TFR than in Helios− TFR. ICOS was increased in Helios+ TFH cells compared with Helios− TFH cells, and ICOS in Helios+ TFH cells was downregulated in SLE. Helios+ TFH cells secreted more IL-21 than Helios− TFH cells, and Helios+ TFH cells from SLE patients had a stronger IL-21 secretion than HCs. Helios+ TFH percentages were negatively correlated with C3 and C4 and positively related to CRP and SLEDAI, and the AUC of Helios+ TFH to distinguish SLE from HC was 0.7959. Helios characterizes circulating TFH cells with enhanced function. Increased Helios+ TFH cells could reflect the autoimmune status of SLE.

**Graphical abstract:**

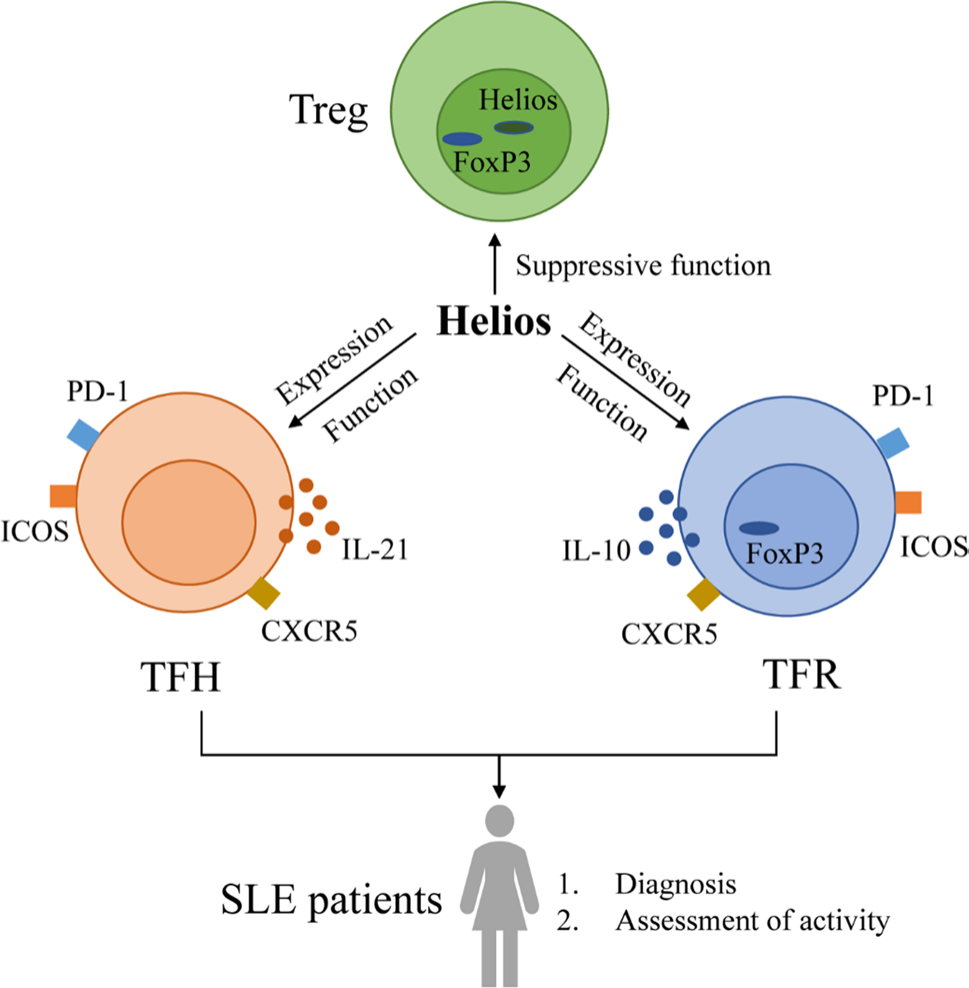

**Supplementary Information:**

The online version contains supplementary material available at 10.1007/s10238-023-01289-6.

## Introduction

Systemic lupus erythematosus (SLE) is a classic autoimmune disease characterized by abnormal immune system activity [1]. Various environmental, genetic, and stochastic factors are involved in the pathogenesis of SLE, leading to the production of autoantibodies that form immune complexes and deposit in various organs, resulting in extensive damage to multiple systems in patients with SLE [2, 3]. B cells play a unique role in the production of autoantibodies, and research has shown that autoreactive B cells originate from activated naïve B cells, which are upregulated in patients with SLE [4, 5]. This upregulation is due to the dysregulation of B-cell selection checkpoints [6, 7].

CD4 T cells are known to be expanded and hyperactive, secreting cytokines and inducing B cells to produce autoantibodies in patients with SLE [8–10]. These cells can be further subdivided into several subgroups, each performing different functions. One such subset is the follicular help T (TFH) cells, which express chemokine receptor 5 (CXCR5), transcription factor B-cell lymphoma 6 protein (Bcl-6), and programmed death receptor-1 (PD-1), and secrete interleukin-21 (IL-21). TFH cells are a distinct CD4 T-cell subset that specializes in helping B cells [9, 11–13]. TFH cells play a crucial role in supporting B-cell selection, differentiation, maturation into memory B cells and plasma cells, and production of immunoglobulin within the germinal center (GC). This process is dependent on the expression of Bcl-6 and the production of IL-21 and other factors [13–16]. In the sanroque mouse model of autoimmunity, enhanced TFH cell development, increased IL-21 production, and abnormal B-cell selection in GCs were observed, resulting in symptoms similar to those of SLE in mice [16–18]. CXCR5 + CD4 + T cells are not only present in lymphoid organs but also in human blood [19]. Recent evidence has suggested that blood CXCR5 + CD4 + T cells may serve as a circulating counterpart of TFH cells, referred to as circulating TFH cells (cTFH) [20]. Our previous study revealed that TFH cells in the peripheral blood of SLE patients were upregulated, and the proportion of TFH subsets was correlated with the percentage of plasmablasts in B cells, anti-double-stranded DNA antibody (anti-dsDNA), and the systemic lupus erythematosus disease activity index (SLEDAI) [21].

GC responses are regulated by follicular regulatory T (TFR) cells, which have specialized functions in suppressing B cell activation mediated by TFH cells. TFR cells express characteristic molecules of both regulatory T (Treg) cells and TFH cells, such as FOXP3 and CXCR5, and secrete interleukin-10 (IL-10) to maintain their regulatory capacity [22, 23]. In mice, the loss of TFR cells results in the accumulation of early plasma cells in GCs, the production of autoantibodies, and the occurrence of autoimmunity [24]. Furthermore, the imbalance of TFH and TFR cells is responsible for the pathogenesis of autoimmune diseases, including SLE and IgG4-related disease[25], as indicated by an increased TFH and TFR cells in IgG4-related disease patients and abnormal TFH/TFR ratio in SLE patients, which is positively correlated with anti-dsDNA antibodies [25, 26].

Helios, encoded by the *IKZF2* gene, is a member of the zinc finger transcription factor family that is expressed in Treg cells and is responsible for their immunosuppressive capacity and stability [27–29]. Helios expression has also been observed in NK cells, where it appears to serve as a marker in the intermediate stage of NK cell differentiation [30, 31]. Although TFR cells were a subgroup of Treg cells, the research of Helios has primarily focused on Treg cells, the expression of Helios in TFR and TFH cells has yet to be fully understood.

This study aims to investigate Helios expression in TFH and TFR cells and to elucidate the functional characteristics of Helios+ TFR and TFH cells. Additionally, the clinical value of Helios+ TFH and TFR cells will be further clarified in patients with SLE.

## Methods

### Bioinformatics analysis

To further understand the expression of Helios (*IKZF2*) in human peripheral blood mononuclear cells (PBMCs), we analyzed the single-cell RNA-Seq database from the NCBI Gene Expression Omnibus (NCBI-GEO) with accession code GSE193096 [32], in which PBMC samples from healthy controls (HCs) were acquired and 16,528 single cells were collected. Furthermore, we utilized the RNA-Seq database with accession code GSE168407 to observe the differential expression of *IKZF2* in TFH cells, Treg cells, and B cells from the tonsils of healthy human donors [33]. Additionally, we analyzed the database with accession code GSE173681 to compare the expression levels of *IKZF2* among conventional T (Tconv) cells, TFH cells, and T regulatory type 1 (TR1) cells from the peripheral blood of type I diabetes patients [34]. We also analyzed the database with accession code GSE181500, which focused on the co-expression profiles of lncRNA and mRNA in CD4 T cells from the peripheral blood of both SLE patients and HCs that were detected by microarray [35].

For the single-cell RNA-Seq database, we utilized the Seurat package (version 4.3.0.1) to perform a standard analysis pipeline. This included selecting the top 2000 highly variable genes and applying a linear transformation, followed by principal component analysis (PCA). We then utilized the uniform manifold approximation and projection (UMAP) method for single-cell cluster visualization. The definition of single-cell clusters was performed using the SingleR package (version 2.2.0) and the hpca.se database. The Seurat function was then used to identify the expression of *IKZF2* mRNA and other marker mRNA in different cell clusters. Furthermore, we analyzed the lncRNA and mRNA co-expression microarray database with GSE181500 and the database with GSE168407 using GEO2R.

### Patients

By the 1982 revised criteria for the classification of systemic lupus erythematosus [36], we enrolled a total of 75 patients with SLE who were treated at Peking University People’s Hospital from December 2021 to June 2022. The calculation of the SLEDAI followed the standard established by Claire et al. in 1992 [37]. SLE patients who were recently diagnosed in our hospital without taking glucocorticoids or immunosuppressant’s were defined as new-onset [38], and 75 SLE patients were divided into 13 new-onset patients and 62 non-new-onset patients according to the disease process. Additionally, 62 HCs were selected as controls, and individuals suffering from cancer, infection, systemic diseases, and other immune diseases were excluded. Patients treated with immunosuppressants over a two-week period were also excluded. The peripheral blood of all or randomly selected patients and HCs were analyzed in different experiments, and there was no significant difference in age and gender distribution between SLE patients and HCs.

### Cell culture

In this study, we obtained whole blood from 15 patients with SLE and 15 HCs, and PBMCs were extracted via Ficoll separation (Dakewei, Shenzhen, China). PBMCs (5 × 10^5^) were then washed twice with PBS and incubated in 96-well plates. The culture medium comprised of RPMI 1640 medium with 10% fetal bovine serum, with the addition of ionomycin (50 ng/ml, InvivoGen, San Diego, CA, USA), phorbol myristate acetate (PMA, 1 μg/ml, InvivoGen), and brefeldin A (BFA, 1:1000, Biolegend, San Diego, CA, USA). Ionomycin and PMA were used to stimulate TFH cells to secrete IL-21 and TFR cells to secrete IL-10, while BFA was used to inhibit the expression of IL-21 and IL-10 to extracellular. Cells were then cultured for 5 h at 37 °C in an atmosphere of 5% carbon dioxide and harvested for flow cytometry analysis.

### Flow cytometry

PBMCs were acquired, and fluorescence antibodies against human CD4-APC-Cy7 (Biolegend, isotype: mouse IgG2b, κ, clone: OK74, cat: 317418) or CD4-PE (Biolegend, isotype: mouse IgG2b, κ, clone: OKT4, cat: 317410), CXCR5-PECy7 (Biolegend, isotype: mouse IgG1, κ, clone: J252D4, cat:356924), PD-1-PerCP-Cy5.5 (Biolegend, isotype: mouse IgG2b, κ, clone: A17188B, cat: 621613), and ICOS-APC-Cy7 (Biolegend, isotype: Armenian Hamster IgG, clone: C398.4A, cat: 313529) were used for cell surface staining. After half an hour, the cells were washed twice with PBS. A transcription factor staining buffer kit (TONBO, San Diego, CA) was used for intracellular staining. Fluorescence antibodies against FOXP3-APC (Invitrogen, isotype: rat IgG2a, κ, clone: PCH101, cat: 17-4776-42), Helios-FITC (Biolegend, isotype: Armenian Hamster IgG, clone: 22F6, cat: 137214), IL-10-PerCP-Cy5.5 (Biolegend, isotype: rat IgG1, κ, clone: JES3-907, cat:501418) and IL-21-PE (Invitrogen, isotype: mouse IgG1, κ, clone: 3A3-N2, cat: 12-7219-41) were used for intracellular staining. Then, the cells were suspended in PBS and analyzed by a FACSCanto cytometer (BD Biosciences, San Jose, CA, USA).

### Clinical parameter measurement

Whole blood cell and lymphocyte counts were measured by a Sysmex XE-2100 (TOA Medical Electronics, Kobe, Japan). IL-21 was analyzed by an ELISA kit from Biolegend (San Diego, CA, USA) according to the manufacturer’s instructions. TGF-β1 was measured by an ELISA kit from MABTECH (MABTECH, Sweden) according to the manufacturer’s instructions. IL-10, interferon-γ and tumor necrosis factor-α (TNF-α) were measured using the Aimplex Human IL-2/IL-4/IL-6/IL-10/TNF-α/interferon-γ 6-plex kit (Quantobio, Beijing, China) according to the manufacturer’s instructions. C-reactive protein (CRP) was detected by i-CHROMA (Boditech Med Inc., Chuncheon, Korea). C3 and C4 were measured by AU5832 automatic biochemistry analyzer (Beckman Couler Inc., CA, USA).

### Statistics

GraphPad Prime 5.5 software (GraphPad, La Jolla, CA, USA) was used to analyze the data. Student’s t test was used to analyze the data between two groups when they met a normal distribution or the Mann‒Whitney test was used for skewed data. When more than two groups, one-way-ANOVA, and multiple comparison tests were used. The proportion results were compared using the chi-square test. A receiver operating characteristic (ROC) curve was used to evaluate the diagnostic values of indicators, and the area under the curve (AUC) was calculated. Spearman’s correlation analysis was conducted to evaluate the relationship of cell subsets with laboratory indicators.

## Results

### Helios was mainly distributed in T cells and NK cells and could express in TFH cells in humans.

**Fig. 1:**
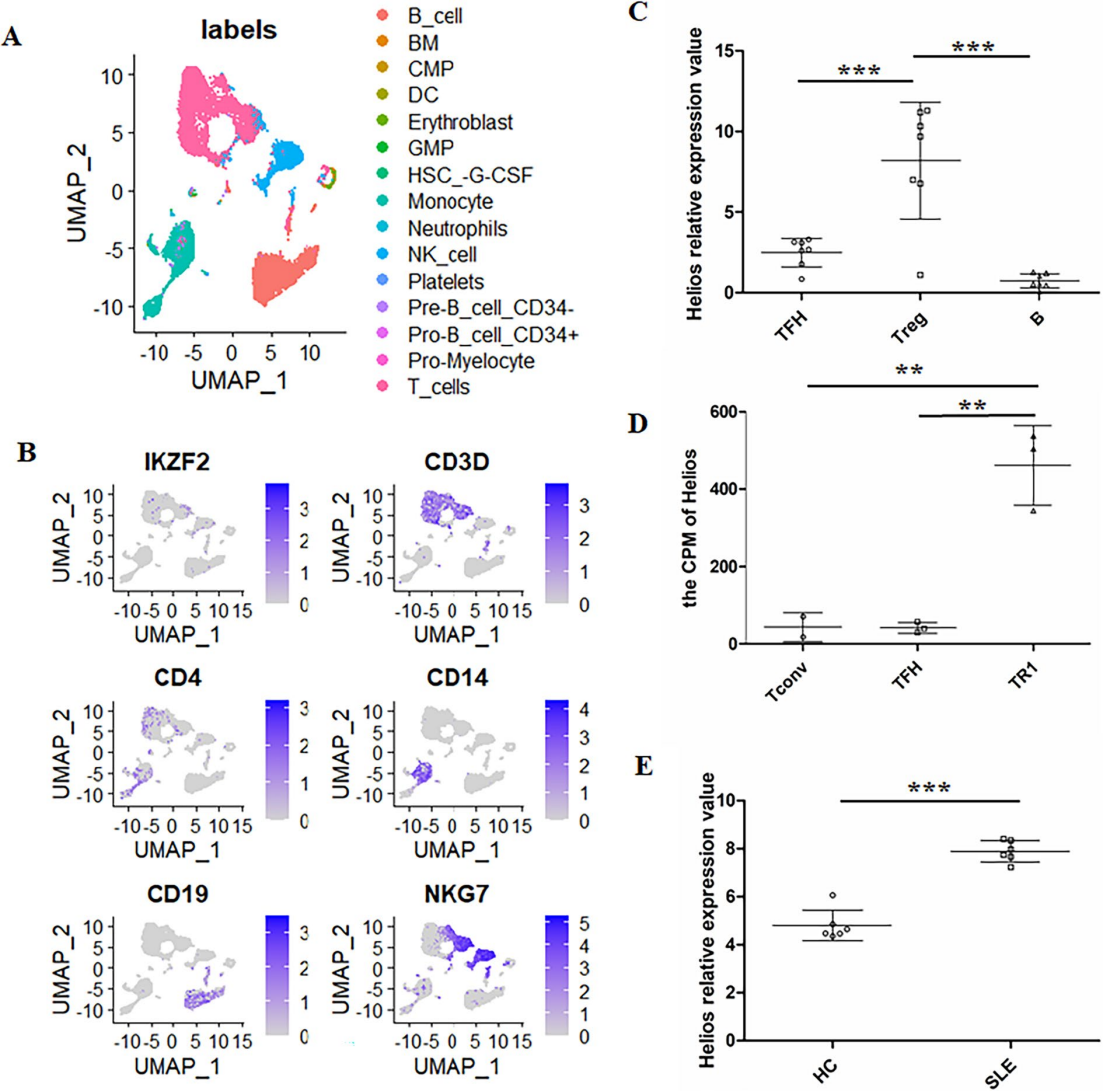
Bioinformatics analysis of *IKZF2* mRNA expression in different cells. The single-cell RNA-Seq database with accession code GSE193096 was analyzed to observe the mRNA expression of *IKZF2* in PBMCs from HCs (*n* = 2). In addition, *IKZF2* mRNA was also investigated in the RNA-Seq database with GSE168407 in TFH cells, Treg cells, and B cells from tonsil of HCs (*n* = 7), and database with GSE173681 in conventional T (Tconv) cells (*n* = 2) and TFH cells (*n* = 3) and T regulatory type 1 (TR1) cells (*n* = 3) from the peripheral blood of type I diabetes patients. lncRNA and mRNA co-expression microarray data with accession code GSE181500 in CD4 T cells from the peripheral blood of SLE patients (*n* = 6) and HCs (*n* = 6) were analyzed to observe the change in *IKZF2* in SLE patients. **a** UMAP plots showing cell clusters in the dataset with GSE193096. **b** UMAP plots showing the expression of *IKZF2*, *CD3D*, *CD4*, *CD14*, *CD19*, and *NKG7* in different cell clusters in the dataset with GSE193096. **c** The comparison of *IKZF2* mRNA among TFH cells, Treg cells, and B cells from the tonsil of HCs in the database with GSE168407. **d** The comparison of *IKZF2* mRNA among Tconv cells, TFH cells, and TR1 cells from the peripheral blood of type I diabetes patients in the database with GSE173681. **e** The comparison of *IKZF2* in CD4 T cells from SLE patients and HCs in the dataset with GSE181500. The results are presented as the mean with SD. ** *P* < 0.01, *** P < 0.001

To investigate the expression of *IKZF2* mRNA in human blood, we analyzed the single-cell RNA-Seq database from NCBI-GEO (accession code GSE193096). Initially, different cell clusters were identified **(**Fig. 1a) [32], and the primary clusters such as T cells, B cells, NK cells, and monocytes were determined using the Seurat package. The UMAP plots revealed that *IKZF2* mRNA was predominantly expressed in CD3 + T cells and NKG7 + NK cells, with minimal expression in CD19 + B cells and negligible expression in CD14 + monocytes (Fig. 1b) [32]. Furthermore, we aimed to investigate the exact expression of *IKZF2* in lymphocyte subsets. Therefore, we analyzed the expression of *IKZF2* in B cells, TFH cells, and Treg cells in the tonsil of HCs. Our observations indicated that the expression of *IKZF2* mRNA was significantly higher in Treg cells compared to TFH and B cells. While there was a tendency for higher expression of *IKZF2* mRNA in TFH cells than B cells, and the difference was not significant (Fig. 1c) [33]. Moreover, the analysis of the PBMC database from type I diabetes patients revealed that TFH cells exhibited a definite expression of *IKZF2* mRNA, although the expression level of *IKZF2* in TFH cells was significantly lower than that in T regulatory type 1 (TR1) cells (Fig. 1d) [34]. Bioinformatic analysis of cell subsets from the germinal center and peripheral blood of humans supported the notion that TFH cells are capable of expressing *IKZF2* mRNA, albeit at a relatively low level. Besides, to preliminarily identify differences in Helios expression between SLE patients and HCs, we analyzed lncRNA and mRNA co-expression microarray databases from SLE patients and HCs. The results indicated that Helios expression was significantly higher in CD4 T cells sorted from the peripheral blood of SLE patients compared to those sorted from HCs (Fig. 1e) [35].

### Analysis of Helios expression in peripheral blood TFR cells and TFH cells.

**Fig. 2:**
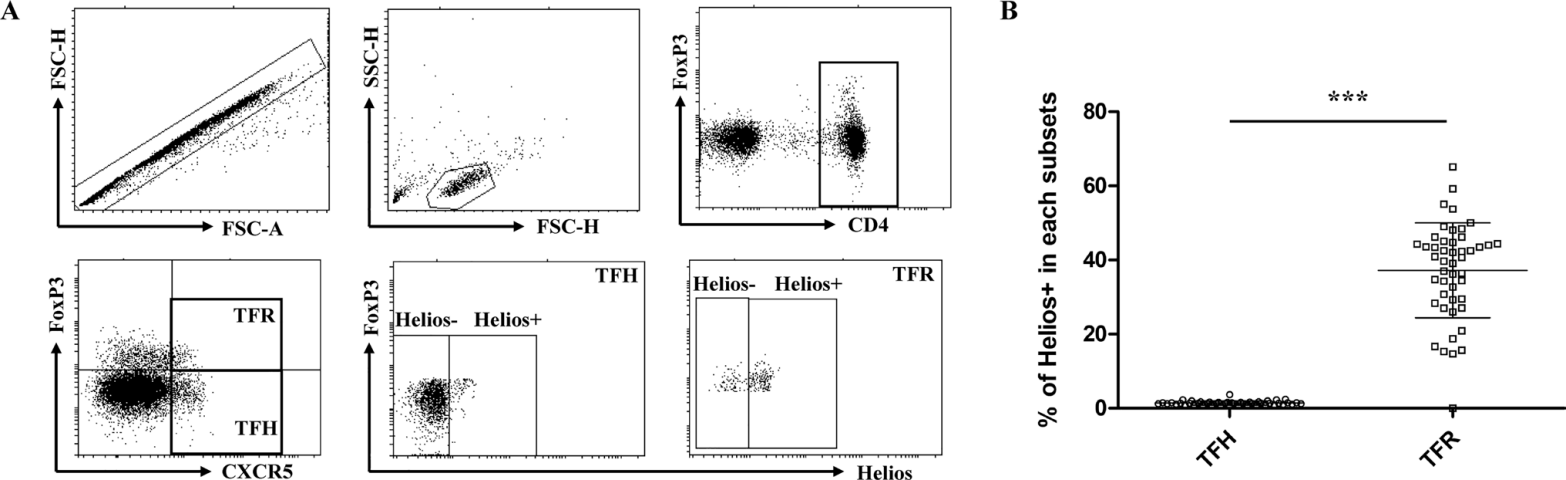
The expression of Helios in circulating TFH and TFR cells. PBMCs from HCs (*n* = 47) were extracted and stained with antibodies against CD4, CXCR5, FOXP3, and Helios. **a** Representative flow cytometry dot plots are shown. Cell subsets were defined as follows: CD4 + FOXP3-CXCR5 + TFH cells and CD4 + FOXP3 + CXCR5 + TFR cells. The expression of Helios was gated according to fluorescence minus one (FMO) controls. **b** The comparison of Helios+ percentages in TFH and TFR cells in HCs. The results are presented as the mean with SD. ***P < 0.001

Based on the results of bioinformatics, we investigated the precise protein expression of Helios in human peripheral blood T-cell subsets, with particular focus on TFH and TFR cells. Using flow cytometry, we examined the expression of Helios in CD4 + FOXP3-CXCR5 + TFH and CD4 + FOXP3 + CXCR5 + TFR cells in HCs (Fig. 2a). Our findings indicated that the proportion of Helios+ cells in TFR cells was significantly higher than that in TFH cells (Fig. 2b).

### Helios+ TFH cells had higher levels of ICOS than Helios-TFH cells

**Fig. 3:**
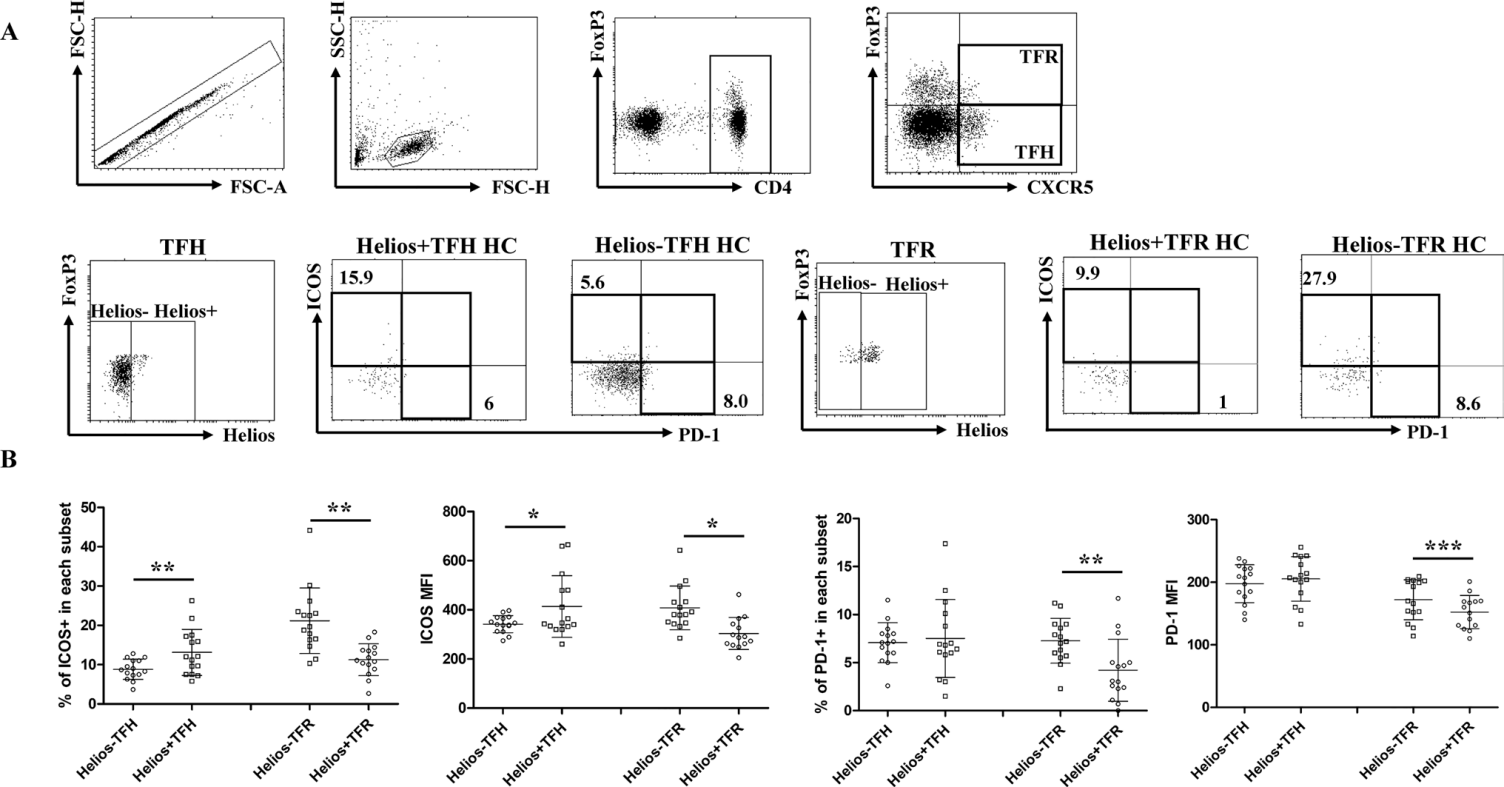
The expression of PD-1 and ICOS in human circulating Helios+ and Helios− TFR and TFH cell subsets. PBMCs from HCs were collected and stained with antibodies against CD4, CXCR5, PD-1, ICOS, FOXP3, and Helios. Helios sorted CD4 + CXCR5 + FOXP3 + TFR and CD4 + CXCR5 + FOXP3- TFH cells into Helios+ and Helios− subsets. PD-1 and ICOS were analyzed in Helios+ and Helios− TFH and TFR cells. **a** Representative flow cytometry dot plots are shown. The numbers indicate percentages of ICOS + or PD-1 + cells, gated according to FMO controls. **b** The comparison of the proportion and mean fluorescence intensity (MFI) of ICOS and PD-1 in TFH and TFR cells in HCs. The results are presented as the mean with SD. *P < 0.05, **P < 0.01, ***P < 0.001

Furthermore, we examined the functional characteristics of Helios+ TFH and TFR cells. PD-1 and ICOS are important surface molecules that play a crucial role in characterizing TFH and TFR functions [39, 40]. We compared the positive percentages and mean fluorescence intensity (MFI) of PD-1 and ICOS between Helios+ and Helios− TFH/TFR subsets in HCs. Our results indicate that in Helios+ TFR cells, both PD-1 and ICOS were downregulated in terms of the positive ratio and MFI compared to Helios-TFR cells. Conversely, Helios+ TFH cells exhibited an upregulation of ICOS percentage and MFI compared to Helios− TFH cells, while there was no significant difference in PD-1 expression between Helios+ and Helios− TFH cells (Fig. 3a, b).

### Helios+ TFH cells had a stronger ability to secrete IL-21

**Fig. 4:**
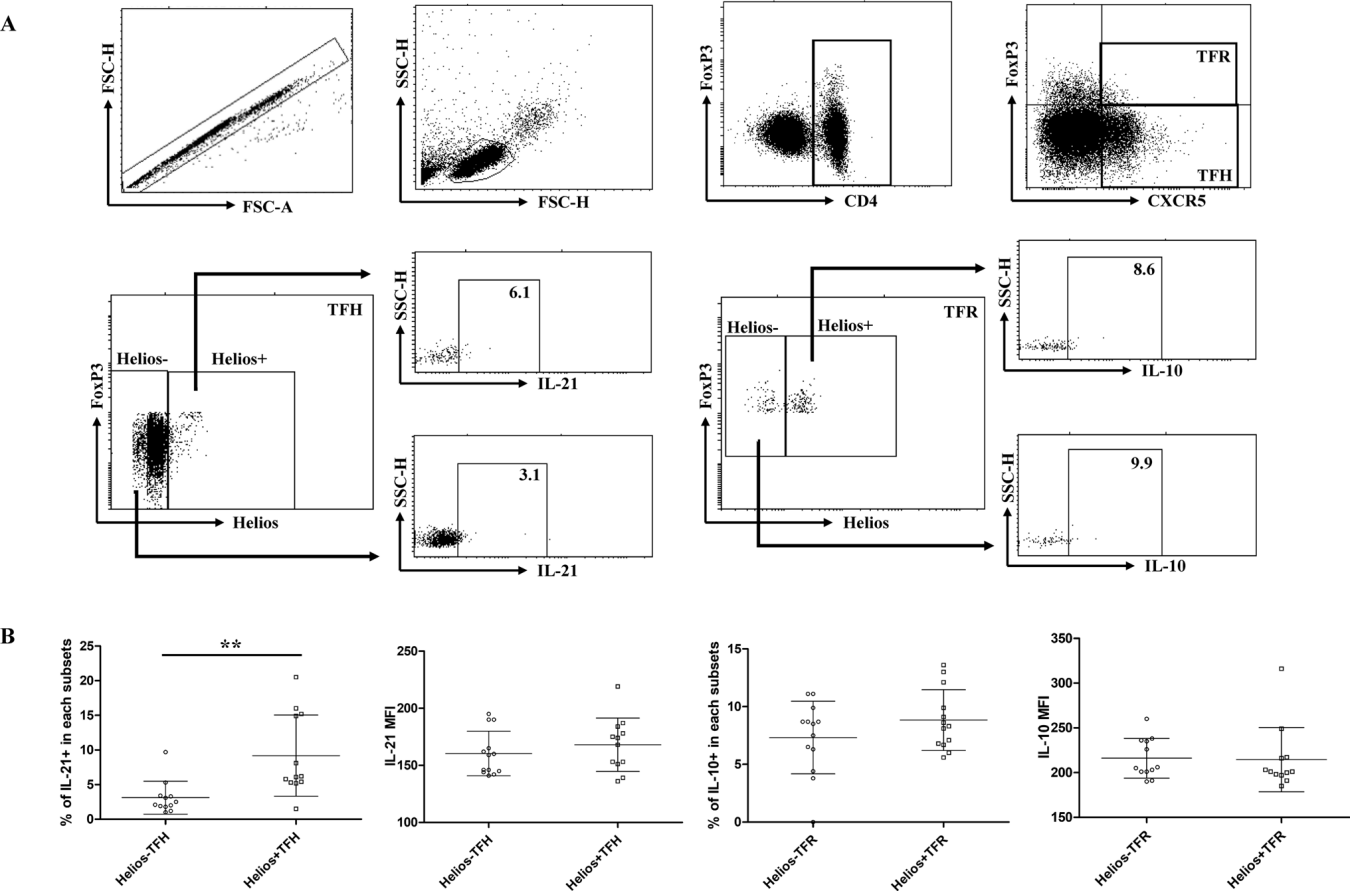
Detection of the ability of Helios+ and Helios-TFH and TFR cells to secrete cytokines. PBMCs from HCs were collected and cultured with PMA, ionomycin, and BFA for 5 h. The ability of Helios+ and Helios− TFH cells and TFR from HCs to secrete IL-21 or IL-10 was assessed by in vitro cell culture and intracellular staining. **a** Representative flow cytometry dot plots are shown. The numbers indicate IL-21- or IL-10-positive percentages, gated according to FMO controls. **b** The comparison of the proportion and MFI of IL-21 in TFH cells and IL-10 in TFR cells. The results are presented as the mean with SD. **P < 0.01

To further investigate the functional characteristics of TFH and TFR cells, we examined their ability to secrete cytokines in HCs. Specifically, we detected the ability of TFH cells to secrete IL-21 and the ability of TFR cells to secrete IL-10 by stimulating PBMC with PMA and ionomycin. Our results indicated that compared to Helios− TFH cells, Helios+ TFH cells had a higher proportion of IL-21 + cells after stimulation, although the MFI difference in IL-21 was not significant. Additionally, we found that the ability of Helios+ TFR cells to secrete IL-10 was not significantly different from that of Helios− TFR cells (Fig. 4a, b).

### Increased Helios+ TFH cells had enhanced IL-21 secretion capacity in patients with SLE

**Fig. 5:**
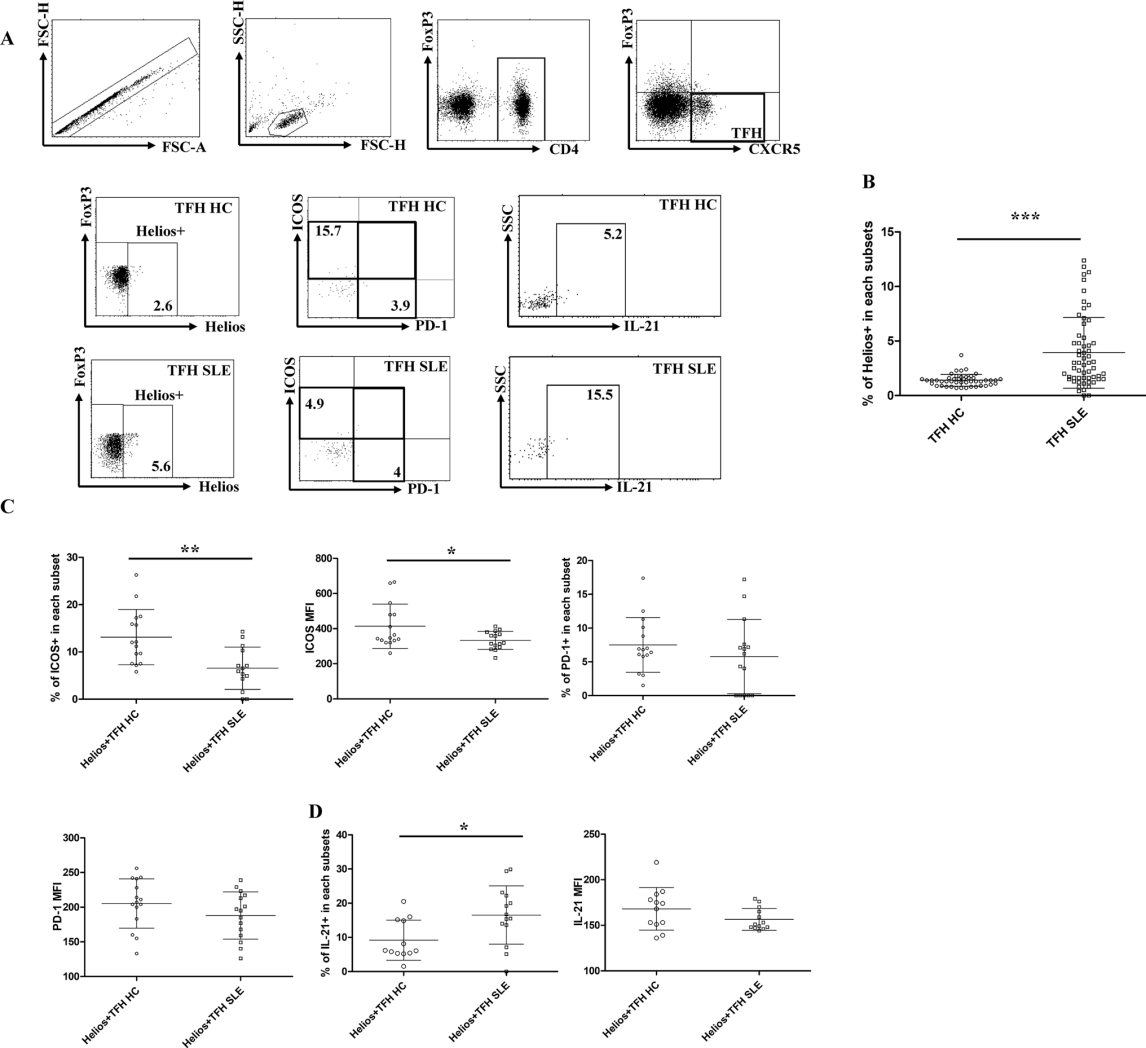
Comparison of surface markers and IL-21 secretion of Helios+ TFH cells in the peripheral blood of SLE patients and HCs. PBMCs from SLE patients and HCs were collected, and Helios+ TFH cells were gated and analyzed between SLE patients and HCs. PD-1 and ICOS expression and IL-21 secretion were further analyzed in Helios+ TFH cells. **a** Representative flow cytometry dot plots are shown. The numbers indicate ICOS + , PD-1 + , or IL-21 + percentages in Helios+ TFH cells in SLE patients and HCs, gated according to FMO controls. **b** The comparison of Helios+ percentages in TFH cells between SLE patients and HCs. **c** The comparison of ICOS + and PD-1 + percentages in Helios+ TFH cells and the MFI of ICOS and PD-1 in Helios+ TFH cells between SLE patients and HCs. **d** The comparison of the proportion and MFI of IL-21 + cells in Helios+ TFH cells between SLE patients and HCs. The results are presented as the mean with SD. *P < 0.05, **P < 0.01, ***P < 0.001

To investigate the clinical significance of Helios+ TFH and TFR cells, we collected peripheral blood from HCs and SLE patients and detected relevant cell subsets using flow cytometry. Demographic and clinical information was presented in Supplementary Table 1. Our results revealed a significant increase in Helios+ TFH cells in the peripheral blood of SLE patients (Fig. 5a, b). In addition, the proportion and MFI of ICOS + subsets in Helios+ TFH cells were decreased in SLE patients. However, there was no significant difference in PD-1 levels in Helios+ TFH cells between the two groups (Fig. 5a, c). Additionally, we found that Helios+ TFH cells from SLE patients had a significantly enhanced ability to secrete IL-21 (Fig. 5a, d). We also investigated Helios+ TFR cells in SLE patients and HCs and found that the proportion of Helios+ TFR cells was significantly upregulated in SLE patients, accompanied by a decrease in the ICOS + percentages (Supplementary Fig. 1). Further analysis of the function of IL-10 secretion in Helios+ TFR cells showed no significant difference in the proportion of IL-10 + cells in Helios+ TFR cells but a significant increase in the MFI of IL-10 + cells in SLE patients than that in HCs (Supplementary Fig. 1).

### Helios+ TFH and TFR cells could reflect the clinical status of SLE patients

We further investigated the relationship between Helios+ TFH and TFR cells in peripheral blood and autoimmunity in SLE patients. As shown in Table 1, there was a significant negative correlation between elevated Helios+ TFH cells and C3 and C4 levels in SLE patients, and Helios+ TFH cells were positively related to SLEDAI and CRP. Helios+ TFR cells, on the other hand, showed a significant positive correlation with serum IgA levels and SLEDAI (Table 1). We also found a significant positive correlation between Helios+ TFR cells and TGF-β1, but no significant correlation between Helios+ TFR cells and serum IL-10 levels (Table 1).

**Table 1:** Correlation analysis of SLE-related clinical indicators with Helios+ percentages in TFH and TFR cells

subsets	SLE indicators	r	*P*
% of Helios+ in TFH	C3	**− 0.3493**	**0.0028**
% of Helios+ in TFH	C4	**− 0.351**	**0.0029**
% of Helios+ in TFH	CRP	**0.3145**	**0.0481**
% of Helios+ in TFH	SLEDAI	**0.3424**	**0.0026**
% of Helios+ in TFH	TNF-α	**− **0.2263	0.0936
% of Helios+ in TFH	IL-21	0.0381	0.7785
% of Helios+ in TFH	IFN-γ	**− **0.1664	0.2162
% of Helios+ in TFH	Disease evolution of SLE (new-onset and non-new-onset)	0.1139	0.3305
% of Helios+ in TFR	TGF-β1	**0.3552**	**0.0105**
% of Helios+ in TFR	IgA	**0.3961**	**0.0033**
% of Helios+ in TFR	SLEDAI	**0.241**	**0.0372**
% of Helios+ in TFR	CRP	**− **0.1838	0.2564
% of Helios+ in TFR	C4	**− **0.2113	0.079
% of Helios+ in TFR	IL-10	**− **0.1251	0.3539
% of Helios+ in TFR	Disease evolution of SLE (new-onset and non-new-onset)	**− **0.1424	0.2230

C3, complement 3. C4, complement 4. CRP, C-reactive protein. IFN-γ, interferon-γ. IgA, immunoglobulin A. IL-10, interleukin-10. IL-21, interleukin-21. r, correlation coefficient. SLEDAI, systemic lupus erythematosus disease activity index. TGF-β1, Transform growth factors-β1. TNF-α, tumor necrosis factor-α. Bold represents correlations with ***P*** values less than 0.05

**Fig. 6:**
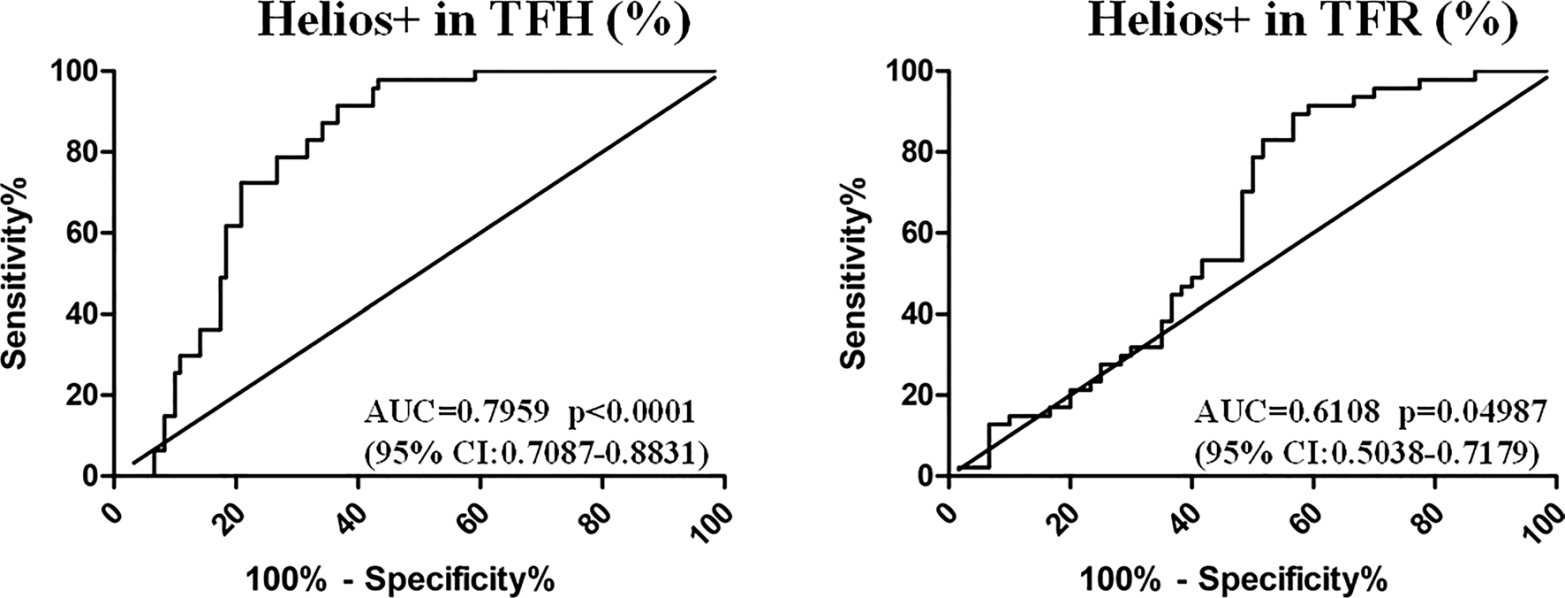
The diagnostic efficacy of Helios-associated TFH and TFR cell subsets in the auxiliary diagnosis of SLE was analyzed by ROC curves. PBMCs from SLE patients (*n* = 60) and HCs (*n* = 47) were collected. ROC curves were used to analyze the diagnostic efficacy of Helios+ percentages in TFH and TFR cells in distinguishing SLE from HCs. The AUCs and 95% confidence intervals are listed

We next generated ROC curves, calculated the AUC, and determined optimal cut-off values using Youden's index. Helios+ TFH cells showed a diagnostic efficacy in distinguishing SLE patients from HCs, with an AUC of 0.7959. On the other hand, Helios+ TFR cells were with an AUC of 0.6108 (Fig. 6). ICOS + percentages in Helios+ TFH cells showed high diagnostic value, with an AUC of 0.7778, while the AUC of other subsets was low (Supplementary Fig. 2). The optimal cut-off for Helios+ percentages in TFH cells was 2.45%, with a sensitivity of 97.87% and specificity of 56.67%, while the optimal cut-off for Helios+ percentages in TFR cells was 49.5% to distinguish SLE from HCs, with a sensitivity of 89.36% and specificity of 43.33% (Supplementary Table 2).

## Discussion

In this study, we investigated the expression and function of Helios in circulating TFH and TFR cells Additionally, we evaluated the clinical significance of Helios+ TFH and TFR cells in SLE. We found that *IKZF2* was specifically expressed in T cells and NK cells, and *IKZF2* was also expressed in TFH cells at a relatively low level. Helios+ TFR cells exhibited lower levels of PD-1 and ICOS than Helios− TFR cells, while Helios+ TFH cells had higher levels of ICOS than Helios− TFH cells. Helios+ TFH cells also secreted more IL-21 than Helios− TFH cells, and Helios+ TFH cells in SLE patients produced more IL-21 than HCs. Accordingly, Helios characterizes circulating TFH cells with enhanced function and increased Helios+ TFH cells could reflect the autoimmune status of SLE patients.

We analyzed multiple sequencing data and found that Helios was predominantly expressed in T cells and NK cells [32]. These findings are consistent with previous reports of Helios expression in T-cell subsets like Treg, as well as in NK cells [27–31]. Furthermore, our bioinformatics analysis revealed that Helios was expressed in TFH cells in both the germinal center and peripheral blood of humans, albeit at lower levels than in Treg cells and TFR cells. This is not unexpected, given that TFR cells are a distinct subset of Treg cells known for their high expression of Helios [41]. Notably, literature on the expression of Helios in TFH cells is scarce. However, a study briefly mentioned this expression by Serre K et al. which supported that Helios was increased in TFH cells in mice with alum-protein vaccines [42]. In this part, sequencing data from patients with type I diabetes were analyzed for *IKZF2* expression in TR1, TFH, and Tconv cells. We found that TR1 cells, as a subset of human peripheral blood Treg cells, were indeed able to express *IKZF2* at the transcription level using only this dataset. However, we were unable to identify other more suitable circulating TR and TFR cellular RNA-seq results from SLE patients, healthy individuals, or other autoimmune patients to support this study. In fact, it is impossible to completely rule out the possible effects of type I diabetes, which is a deficiency of this article. However, we proved the results of the sequencing analysis by flow cytometry, thus guaranteeing the reliability of the final conclusion.

Our study found that ICOS and PD-1 expression was lower in Helios+ TFR cells, while ICOS was higher in Helios+ TFH cells than in Helios− TFH cells. ICOS is a potent functional molecule that facilitates TFH cell-mediated B-cell help [43]. Deficiency in ICOS results in a severe loss of memory B cells and a complete blockade of antigen-specific IgG responses [44]. PD-1 is crucial for T-cell tolerance and is highly expressed in both germinal center and peripheral blood TFH cells [45]. High levels of PD-1 expression on TFH cells are essential for B-cell response and antibody production [46]. Hence, the expression of ICOS and PD-1 is indicative of activated TFH cells and plays a crucial role in facilitating B-cell antibody production. For TFR cells, the absence of PD-1 leads to increased TFR cells in circulation and enhances their in vivo suppression capacity [39]. Additionally, ICOS provides essential costimulatory signals to TFR cells in both peripheral blood and lymph nodes [47]. Our results suggest that Helios+ TFH cells may have a relatively enhanced helper function, and Helios+ TFR cells may also be enhanced due to the downregulation of PD-1 and weakening of the ICOS co-stimulation signal. Furthermore, the relatively enhanced functional phenotype of Helios+ TFH cells was validated by their increased IL-21 secretion. There have reported that Helios could be induced during the activation and proliferation of T cells [48], and Helios+ FoxP3 + cells in mice had a higher level of CD44 expression while Helios-FoxP3 + cells expressed lower CD44 [49]. CD44 and CD69 are both markers of lymphocyte activation [50]. According to our preliminary experimental findings, CD44 and CD69 expressions between Helios+ and Helios− TFH cells in HC were not significantly different (data not shown), so the expression of Helios may not just the reflection of T cell activation. How Helios affects the behavior of TFH cells still needs to be further explored in the future.

Due to the intracellular nature of Helios, we could not sort Helios+ TFH cells for in vitro functional testing, which posed a limitation in our research. However, by combining cell phenotypic characteristics and cytokine secretion function, we can confidently conclude that Helios+ TFH cells with upregualted expression of ICOS and higher secretion of IL-21 exhibit an enhanced functional phenotype.

We found that the expression of Helios in TFH cells was significantly upregulated in SLE patients. Helios+ TFH cells in SLE patients exhibited a lower proportion of ICOS but secreted significantly more IL-21 than HCs. We speculate that the ability of TFH cells to secrete IL-21 may depend on other molecules, such as ROCK2, a Rho kinase [51]. IL-21 secretion by Helios− TFH cells in SLE patients was also significantly lower than that of Helios+ cells (not shown). Helios− TFH cells represent the majority of TFH cells in SLE and exhibit a weakened functional phenotype compared to Helios+ TFH cells. This finding is consistent with our previous study, which demonstrated that expanded TFH cells in SLE are functionally impaired [21].

Our correlation analysis revealed that Helios+ TFH cells were negatively correlated with C4 and C3, which were biomarkers to diagnose and assess disease activity [52]. Besides, Helios+ TFH cells were also positively related to CRP and SLEDAI, a system for assessing disease activity [37], indicating that Helios+ TFH cells also could aid in the diagnosis and evaluation of SLE disease activity. ROC curve results suggested that Helios-related subsets are useful in distinguishing SLE patients from healthy individuals, while their early diagnosis and efficacy evaluation have not been well studied. As for the disease controls were lacked, we could not make sure whether Helios expression levels change in circulating TFH and TFR cells from patients with other autoimmune diseases. To further analyze its diagnostic value in SLE, a larger sample size and sufficient disease controls need to be included in future analyses.

In conclusion, our study focused on the expression of Helios in TFH and TFR cells and found that increased Helios+ TFH cells in SLE were associated with a stronger ability. Additionally, Helios+ TFH cells can serve as a biomarker to reflect the autoimmune status of SLE and aid in its diagnosis.

## Supplementary Information

Below is the link to the electronic supplementary material.

Supplementary file 1 (TIF 443 KB)

Supplementary file 2 (TIF 340 KB)

Supplementary file 3 (DOCX 17 KB)

Supplementary file 4 (DOCX 16 KB)

Supplementary file 5 (DOC 20 KB)

## Data Availability

Data on this study were available from the corresponding authors on a reasonable request.

## Abbreviations

Anti-dsDNA: Anti-double-stranded DNA antibody
AUC: Area under the curve
Bcl6: B-cell lymphoma 6 protein
CPM: Count-per-million
CRP: C-reactive protein
CXCR5: Chemokine receptor 5
FMO: Fluorescence minus one
GC: Germinal center
HCs: Healthy controls
ICOS: Inducible costimulatory
IL-10: Interleukin-10
IL-21: Interleukin-21
NCBI-GEO: NCBI Gene Expression Omnibus
PBMCs: Peripheral blood mononuclear cells
PCA: Principal component analysis
PD-1: Programmer death receptor-1
PMA: Phorbol myristate acetate
ROC: Receiver operating curve
SLE: Systemic lupus erythematosus
SLEDAI: Systemic lupus erythematosus disease activity index
TFH: Follicular help T
TFR: Follicular regulatory T
TGF-β: Transform growth factors-β
Treg: Regulatory T
UMAP: Uniform manifold approximation and projection
