# Supplementary figures and images for: Helios characterized circulating follicular helper T cells with enhanced functional phenotypes and was increased in patients with systemic lupus erythematosus
Source: Clin Exp Med. 2024 Jan 19;24(1):5. doi: 10.1007/s10238-023-01289-6 (PMC10799143; doi:10.1007/s10238-023-01289-6)

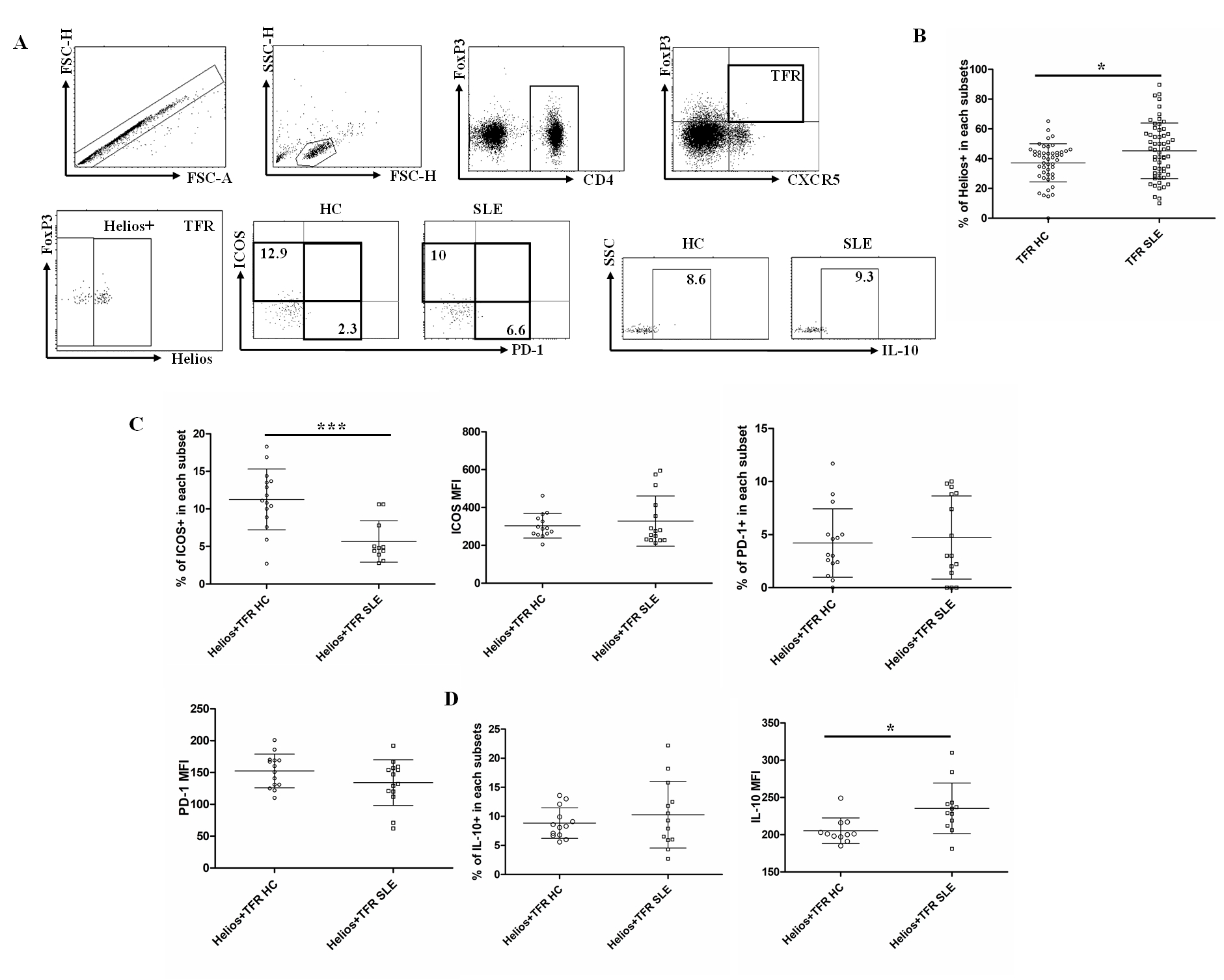

Supplement: Supplementary file 1 — (TIF 443 KB) [file 10238_2023_1289_MOESM1_ESM.tif]

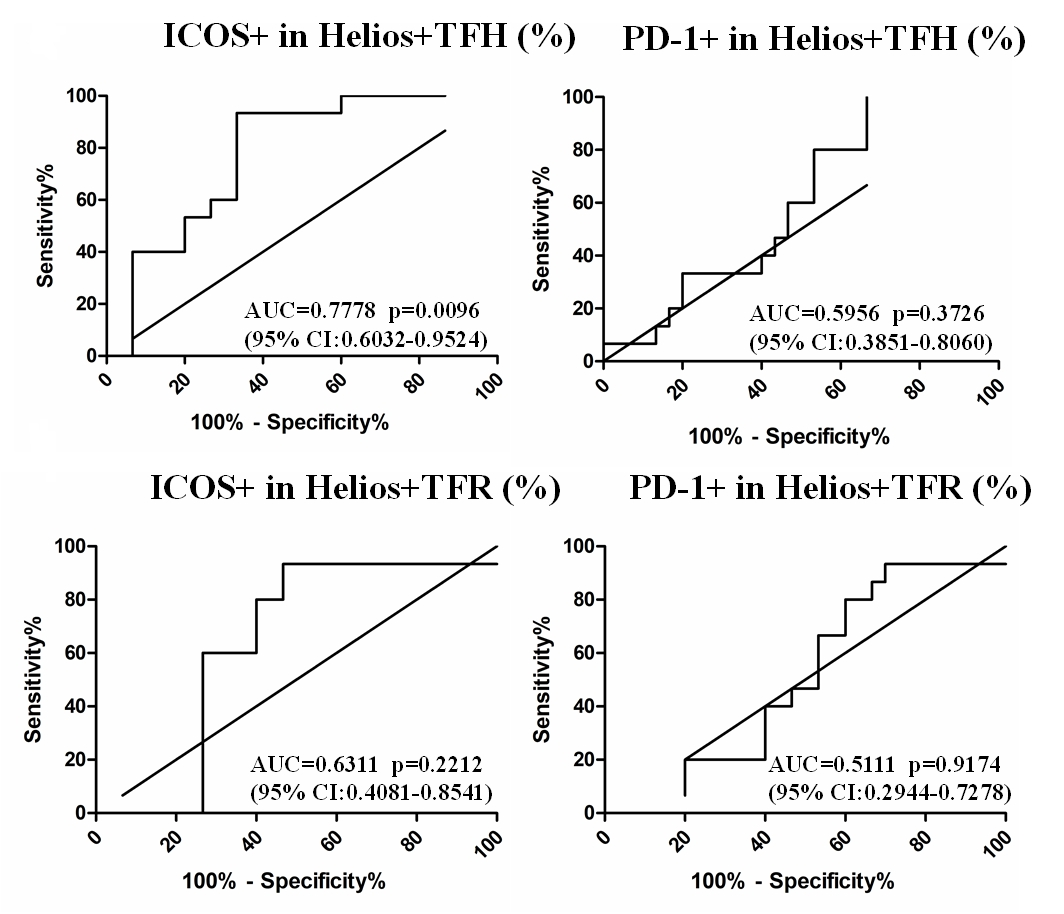

Supplement: Supplementary file 2 — (TIF 340 KB) [file 10238_2023_1289_MOESM2_ESM.tif]
