# Supplementary material for: Helios characterized circulating follicular helper T cells with enhanced functional phenotypes and was increased in patients with systemic lupus erythematosus
Source: Clin Exp Med. 2024 Jan 19;24(1):5. doi: 10.1007/s10238-023-01289-6 (PMC10799143; doi:10.1007/s10238-023-01289-6)
Supplement: Supplementary file 3 — (DOCX 17 KB) [file 10238_2023_1289_MOESM3_ESM.docx]

**Supplementary Figure 1. Analysis and Comparison of Expression and Function of Helios+ TFR Cells in Peripheral Blood of SLE Patients and Healthy Controls.**

Peripheral blood mononuclear cells (PBMC) were collected from SLE patients (n=75) and HCs (n=62), and Helios+ TFR cells were gated and analyzed in both groups. Subsequently, PD-1 and ICOS expressions and IL-10 secretion were analyzed in Helios+ TFR cells.

A. The figure shows representative dot plots obtained through flow cytometry analysis. The numbers indicate the percentage of ICOS+, PD-1+ or IL-10+ cells in Helios+ TFR cells, gated according to fluorescence minus one (FMO) controls.

B. Comparison of Helios+ percentages in TFR between SLE patients (n=60) and HCs (n=47).

C. Comparison of ICOS+ and PD-1+ percentages in Helios+ TFR cells and the mean fluorescence intensity (MFI) of ICOS and PD-1 in Helios+ TFR between SLE patients (n=15) and HCs (n=15).

D. Comparison of the proportion and MFI of IL-10+ cells in Helios+ TFR between SLE patients (n=15) and HCs (n=15).

The results are presented as mean values with standard deviation. *p<0.05; ***p<0.001.

**Supplementary Figure 2. The diagnostic efficacy of ICOS and PD-1 in Helios+ TFH and TFR cells in SLE patients from HC.**

ROC curves were used to analyze the diagnostic efficacy of ICOS+ and PD-1+ percentages in Helios+ TFH and TFR cells in distinguishing SLE patients (n=15) from HCs (n=15). The AUCs and 95% confidence intervals are listed.
