# Supplementary material for: Helios characterized circulating follicular helper T cells with enhanced functional phenotypes and was increased in patients with systemic lupus erythematosus
Source: Clin Exp Med. 2024 Jan 19;24(1):5. doi: 10.1007/s10238-023-01289-6 (PMC10799143; doi:10.1007/s10238-023-01289-6)
Supplement: Supplementary file 4 — (DOCX 16 KB) [file 10238_2023_1289_MOESM4_ESM.docx]

**Supplementary table 1. Demographic and clinical characteristics of the subjects in this study.**

|  | characteristics | SLE | HC | P |
| --- | --- | --- | --- | --- |
| basic characteristics | numbers | 75 | 62 |  |
|  | gender, male/female | 8/67 | 7/55 | 0.91 |
|  | age, year, mean±SD | 42.21±15.19 | 43.9±12.96 | 0.50 |
| disease evolution | new-onset (n) (%) | 13 (17.33%) |  |  |
| clinical manifestations | hematological disorders (n) (%) | 45 (60%) |  |  |
|  | bone damage (n) (%) | 34 (45.33%) |  |  |
|  | skin damage (n) (%) | 22 (29.33%) |  |  |
|  | kidney damage (n) (%) | 19 (25.33%) |  |  |
|  | neurological damage (n) (%) | 9 (12%) |  |  |
|  | joint injury (n) (%) | 8 (10.67%) |  |  |
| medication history | glucocorticoids  (n) (%) | 38 (50.67%) |  |  |
|  | antimalarial drugs  (n) (%) | 50 (66.67%) |  |  |
|  | immunosuppressants (n) (%) | 48 (64%) |  |  |
| laboratory  characteristics | anti-dsDNA, mean±SD | 20.77±32.32 |  |  |
|  | C3, mean±SD, G/L | 0.95±0.25 |  |  |
|  | C4, mean±SD, G/L | 0.26±0.13 |  |  |
|  | CRP, mean±SD, mg/L | 3.31±7.7 |  |  |
|  | ESR, mean±SD, mm/hour | 17.98±17.73 |  |  |
|  | IgA, mean±SD, G/L | 2.49±1.15 |  |  |
|  | IgG, mean±SD, G/L | 12.68±3.39 |  |  |
|  | IgM, mean±SD, G/L | 0.93±0.50 |  |  |
|  | SLEDAI | 7.31±3.34 |  |  |

Anti-ds-DNA antibody, anti-double stranded DNA antibody. C3, complement 3. C4, complement 4. CRP, C-reactive protein. ESR, erythrocyte sedimentation rate. Ig, immunoglobulin. SLEDAI, systemic lupus erythematosus disease activity index.
