# Supplementary material for: Helios characterized circulating follicular helper T cells with enhanced functional phenotypes and was increased in patients with systemic lupus erythematosus
Source: Clin Exp Med. 2024 Jan 19;24(1):5. doi: 10.1007/s10238-023-01289-6 (PMC10799143; doi:10.1007/s10238-023-01289-6)
Supplement: Supplementary file 5 — (DOC 20 KB) [file 10238_2023_1289_MOESM5_ESM.doc]

**Supplementary Table 2. The cut-off values for Helios+ TFH and TFR cells in distinguishing SLE from healthy individuals.**

| **Subsets** | **Cut-off values** | **Sensitivity** | **Specificity** |
| --- | --- | --- | --- |
| **% of Helios+ in TFH** | 2.45% | 97.87% | 56.67% |
| **% of Helios+ in TFR** | 49.5% | 89.36% | 43.33% |
| **% of ICOS+ in Helios+TFH** | 7.15% | 93.33% | 66.67% |
| **% of ICOS+ in Helios+TFR** | 5.45% | 93.33% | 53.33% |
| **% of PD-1+ in Helios+TFH** | 0.75% | 100% | 33.33% |
| **% of PD-1+ in Helios+TFR** | 6.2% | 80% | 40% |
